# Short-Term Responses in Maximum Quantum Yield of PSII (Fv/Fm) to *ex situ* Temperature Treatment of Populations of Bryophytes Originating from Different Sites in Hokkaido, Northern Japan

**DOI:** 10.3390/plants5020022

**Published:** 2016-04-26

**Authors:** Annika K. Jägerbrand, Gaku Kudo

**Affiliations:** 1 Faculty of Environmental Earth Science, Hokkaido University, Sapporo, 060-0810 Hokkaido, Japan; gaku@ees.hokudai.ac.jp; 2 The Swedish National Road and Transport Research Institute, Box 55685, SE-102 15 Stockholm, Sweden

**Keywords:** stress, indicator, terrestrial, mosses, heat, cold

## Abstract

There is limited knowledge available on the thermal acclimation processes for bryophytes, especially when considering variation between populations or sites. This study investigated whether short-term *ex situ* thermal acclimation of different populations showed patterns of site dependency and whether the maximum quantum yield of PSII (Fv/Fm) could be used as an indicator of adaptation or temperature stress in two bryophyte species: *Pleurozium schreberi* (Willd. ex Brid.) Mitt. and *Racomitrium lanuginosum* (Hedw.) Brid. We sought to test the hypothesis that differences in the ability to acclimate to short-term temperature treatment would be revealed as differences in photosystem II maximum yield (Fv/Fm). Thermal treatments were applied to samples from 12 and 11 populations during 12 or 13 days in growth chambers and comprised: (1) 10/5 °C; (2) 20/10 °C; (3) 25/15 °C; (4) 30/20 °C (12 hours day/night temperature). In *Pleurozium schreberi*, there were no significant site-dependent differences before or after the experiment, while site dependencies were clearly shown in *Racomitrium lanuginosum* throughout the study. Fv/Fm in *Pleurozium schreberi* decreased at the highest and lowest temperature treatments, which can be interpreted as a stress response, but no similar trends were shown by *Racomitrium lanuginosum*.

## 1. Introduction

There is limited knowledge available on the thermal acclimation processes for bryophytes, especially when considering variation among and between populations, sites and time scales [1,2,3]. Thermal acclimation in terms of performance adjustments to temperature change enables plants to maintain photosynthesis under differing temperature regimes [4]. Consequently, thermal acclimation is important for the survival and growth of individuals and may also determine the geographical boundaries of species. Transplantation studies have shown that bryophytes may exhibit higher net assimilation rates under a range of different temperatures, which could indicate higher acclimation potential [5], but also that acclimation in samples transplanted to field conditions may be reduced and result in high mortality and lower growth rates [3].

A four-week study involving *ex situ* temperature treatment of the bryophyte *Pleurozium schreberi* (Willd. ex Brid.) Mitt. originating from eight sites at differing altitudes in Hokkaido, Japan, showed that the responses were dependent upon the sampling site and that treatment effects differed between sites [2]. Those results raised important concerns regarding the general validity of temperature responses in experiments performed on a single species or a limited number of populations/sites of bryophytes.

The main aim of this study was therefore to investigate whether short-term thermal acclimation of different populations of bryophytes showed similar patterns of site dependencies as found previously in *P. schreberi* [2]. However, since bryophytes are known for exhibiting inter-species differences in their temperature optimum for growth [6], it was decided to include one more species (*Racomitrium lanuginosum* (Hedw.) Brid.) in this study to make the results comparable on an inter-species level.

For short-term responses, growth rate or shoot length increases are not possible to measure and instead Fv/Fm, a measurement of the maximum quantum yield of PSII performed on dark-adapted samples, was used. Fv/Fm is a sensitive indicator of plant photosynthetic performance, but lower values may also indicate stress and/or photoinhibition [7], or indicate downregulation of photosynthesis. Chlorophyll fluorescence of plants has been used as an indicator of environmental stress and temperature stress, e.g., [8], but has not received as much attention in bryophytes. However, a few studies have investigated the relationship between Fv/Fm and desiccation [9,10,11]. In the present study, it was assumed that Fv/Fm values reflected the capacity to adapt to the stress conditions of the new temperature regime *ex situ* but that Fv/Fm also, at least partly, reflected pre-treatment conditions.

A second aim was to confirm whether Fv/Fm could be used as a short-term indicator of adaptation or temperature stress in bryophytes. Decreased Fv/Fm values compared with before the start of the experiment were taken to indicate stress due to decreased photosynthetic performance, while similar or higher values were taken to indicate adaptation capability to *ex situ* temperature manipulation. Based on this, the starting hypothesis was that species with significant site-specific Fv/Fm have lower acclimation capacity to different temperature conditions due to lower plasticity (can be regarded as specialists), while the opposite occurs in species with little significant site-specific variation in Fv/Fm due to higher plasticity (can be regarded as generalists) and thus they have a higher acclimation potential.

## 2. Results

**Figure 1:**
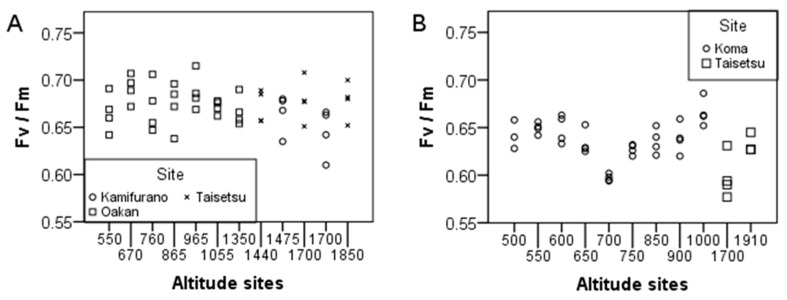
Fv/Fm before the experiment for different altitudinal sites and their areas in: (**A**) *P. schreberi* and (**B**) *R. lanuginosum*. For statistical significance, see Table 1.

There were no significant differences in Fv/Fm between sites of *P. schreberi* prior to the experiment, while significant site differences were found in *R. lanuginosum* (Table 1 and Figure 1). Site differences in Fv/Fm before the temperature treatment were not clearly distinguishable in *P. schreberi* while site differences of *R. lanuginosum* were observable and seemed to be related to both altitudes and the two collection sites (Figure 1).

**Figure 2:**
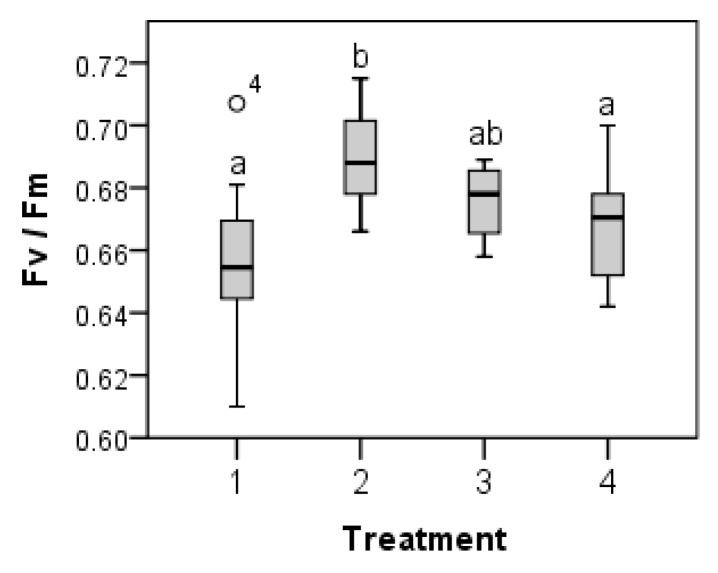
Box plots of Fv/Fm in *P. schreberi* from 12 different sites, in response to short-term (13 days) temperature treatment *ex situ*. Different letters indicate significant difference (determined by Fisher’s PLSD *post-hoc* test). Treatments in day/night temperatures: 1 = 10/5 °C; 2 = 20/10 °C; 3 = 25/15 °C; 4 = 30/20 °C. The median in the box plot is indicated by the horizontal line in the box, the box indicates the 25th and 75th percentiles and the bars indicate the 10th and 90th percentiles. n = 12 (sites). Site effects were non-significant (Table 2).

**Figure 3:**
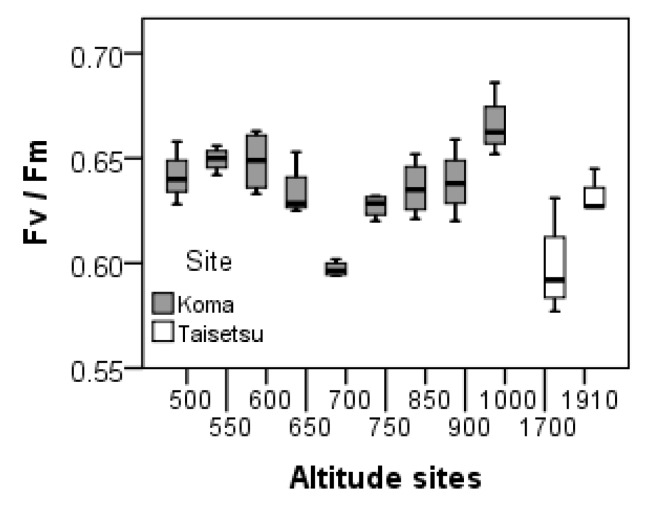
Box plots of Fv/Fm in *R. lanuginosum* from 11 different sites in Japan, after being exposed to short-term (12 days) temperature treatment *ex situ*. The median in the box plot is indicated by the horizontal line in the box, the box indicates the 25th and 75th percentiles and the bars indicate the 10th and 90th percentiles. Samples from Mt. Koma (altitude 500–1000 m above sea level) and Taisetsu (altitude 1700 and 1910 m above sea level). n = 4 (3 for altitude site 550). Effects of temperature treatments were non-significant (Table 3).

*P. schreberi* showed significant responses in Fv/Fm to the temperature treatments, with the highest Fv/Fm seen in treatment 2 with 20/10 °C (day/night) temperature conditions (Table 2 and Figure 2). *P. schreberi* did not show any significant differences in Fv/Fm due to site differences (Table 2) after the short-term temperature treatments. However, *R. lanuginosum* showed the opposite response, with significant differences in Fv/Fm between the 11 sites, but no significant differences in response to the temperature treatments (Table 2 and Figure 3). No significant differences were found in Fv/Fm before and after short-term temperature treatment (Table 3) in either species.

## 3. Discussion

The two species were found to have different acclimation potential in their photosynthetic performance and stress tolerance when exposed to different temperatures *ex situ* after only 12–13 days. In *P. schreberi*, Fv/Fm prior to the experiment was quite similar between populations, while in *R. lanuginosum* Fv/Fm varied between sites from the beginning and these original differences were still present after the short-term experiment. Thus, *P. schreberi* seemed to adapt more quickly than *R. lanuginosum*. *P. schreberi* showed lower Fv/Fm values at the lowest and highest temperatures, indicating temperature stress. This confirms our hypothesis that significant site-specific variation may cause lower acclimation capacity, while species with little significant site-specific variation may have higher acclimation capability.

Regarding the first aim of confirming whether populations of bryophytes show site dependencies to temperature treatments, as found previously for *P. schreberi* [2], there were no significant site differences before or after the experiment in *P. schreberi*, while site dependencies were clearly shown by *R. lanuginosum*. Differences in growing conditions from the original habitat, species-specific ecology and plasticity may explain the differing photosynthetic responses to the temperature treatments in the two species. *P. schreberi* generally grows in shaded conditions such as the forest floor and is a temperate species, increasing productivity with ambient temperature to a certain degree. Since the temperature optimum for net photosynthesis in bryophyte species correlates well with the mean temperature of the habitat during the growing season [1], it is not surprising that *P. schreberi* acclimated well to some of the temperatures in the growth chambers. In contrast, *R. lanuginosum* is commonly found in exposed habitats in colder or alpine areas and it has a temperature optimum for net photosynthesis of 5 °C regardless of latitude [12], indicating very low acclimation potential [1]. Differing acclimation potential among species and populations over time and in degree of response may explain why, e.g., polar bryophytes exhibit high or low acclimation potential and tropical bryophytes show lower growth rates after being transplanted into field conditions. Differences in acclimation between populations of bryophytes have been reported previously [13]. In our previous study [2], both shoot length increase and Fv/Fm response of *P. schreberi* to different temperature conditions were affected by the site of origin even after four weeks of growth in the growth chambers [2]. The somewhat contradictory results compared with this study may depend on several factors, such as different timescales and differences in the temperature treatments applied. The previous study examined responses to control (20/10 °C), press and pulse treatments, while this study only included four different temperature regimes. For control treatments, values of Fv/Fm were around 0.65–0.73 in [2], which is somewhat higher than, e.g., in treatment 1 in this study, but the values here varied greatly between sites.

One aspect that might affect the results reported in this study is the time of plant collection relative to the start of the experiments. Glime [14] showed that bryophytes collected at different times during the year showed different temperature optima for growth and that bryophytes collected later in the year ceased growth after two to three weeks, which was perhaps predetermined by an internal physiological clock. In this and our previous study [2], the samples used were collected in the same period of the year, thus eliminating the direct effects of time of sampling. However, it is possible that populations along altitude gradients have adjusted to different temperature cycles around the year, although such patterns in growth and temperature dependencies would be difficult to study without collecting samples from the same sites, but at different times of the year, and exposing them to similar conditions.

Future studies could include analysis of bryophytes grown in growth chambers for a longer period, in combination with a reciprocal transplant study of plant material from the same sites and areas, as done in this study, to reveal more about the temperature response patterns in Fv/Fm shown by the bryophytes in this study.

## 4. Experimental Section

A previous study investigated the genetic structure of *P. schreberi* and *R. lanuginosum* from the study sites [15]. The two bryophytes used in this study are common and widely distributed species, e.g., *P. schreberi* is circumpolar and *R. lanuginosum* is cosmopolitan. They have a different temperature optimum for growth. *P. schreberi* is a temperate species common in coniferous forests, with optimal temperature for growth within the range 15–25 °C [6], whereas *R. lanuginosum* is a cold-adapted species common in alpine regions, showing a photosynthetic optimum at 5 °C [16].

Plant collection took place at a wide range of different altitudes in Hokkaido, Japan. Altitudinal gradients are appropriate for comparisons between populations adapted to various environmental conditions, since the slopes provide natural temperature variations at relatively close geographical distances. Samples of *P. schreberi* were collected at 12 sites in three areas and samples of *R. lanuginosum* were collected at 11 sites in two areas, all in June 2007. For further details see Table 4, [15] or [2]. All samples were kept in dry, dark and cool conditions from collection until the first Fv/Fm measurements (prior to the experiment) and thereafter until the experiment started (on 5–6 July 2007). 

From each site, five moss shoots from each of four samples were randomly chosen and cut to 4 cm (*P. schreberi*) or 2 cm (*R. lanuginosum*) and Fv/Fm measurements were made before the experiment started. Shoots were then individually marked and placed into small transparent plastic bags (9 cm × 15 cm), and assigned to one of four temperature treatments. Samples in bags were fully hydrated with de-ionized water and suspended in a natural position in growth chambers on 5 July for *P. schreberi*, and on 6 July for *R. lanuginosum*. The growth chambers simulating different temperature conditions had 12 h day/night temperature cycle and photoperiod (Table 5). Temperatures in the growth chambers were (1) 10/5 °C; (2) 20/10 °C; (3) 25/15 °C; (4) 30/20 °C (day/night). These temperatures were chosen to represent both the low temperature conditions occurring at high altitudes and the high temperatures that are realistic in the Japanese summer climate at the lowest altitudes. For further information see [2]. Light conditions inside the growth chambers were 15–30 µM m^–2^ s^–1^ measured with a quantum photometer (Li-250, Li-Cor, Lincoln, NE, USA), which is generally considered a low value. However, *P. schreberi* grows underneath the vegetation canopy at all sites and was therefore likely to be shade-adapted. The light responses were assumed to be similar to those of bryophyte species growing in forests. For example, several bryophyte species show a light saturation point of less than 30 µM m^–2^ s^–1^ in laurel evergreen forests [17]. Furthermore, since Fv/Fm is also an indicator of stress to high light conditions, it was considered important not to induce photoinhibition. Samples were sprayed with de-ionized water at regular intervals throughout the experimental period to ensure that drought or lack of CO_2_ did not limit growth.

Before the start of the experiment and after 12 (*R. lanuginosum*) and 13 (*P. schreberi*) days, shoots were carefully removed from plastic bags and put in bunches to increase the surface area of chlorophyll fluorescence measurements (from each treatment and site for the treated samples), and placed in leaf clips for two hours of dark adaptation. Maximum quantum of PSII (Fv/Fm) was measured with a mini-PAM (miniaturized pulse amplitude–modulated photosynthesis yield analyzer; Walz, GmbH, Effeltrich, Germany). Calculations followed [18]. In one sample (*R. lanuginosum*, Mt Koma 550 m above sea level), the measurement on Fv/Fm was later determined to be wrong and was not included in the analyses.

The data were log-transformed to meet normality assumptions and then investigated for differences in Fv/Fm in response to either site (before and after) or temperature treatment (after). Since there was only one value of Fv/Fm for each site and treatment after the experiment was finished, separate one-way ANOVAs were performed for *P. schreberi* and *R. lanuginosum*. Significant differences for *P. schreberi* to treatments were subsequently determined with Fisher’s PLSD *post-hoc* test. We used t-test to investigate differences in Fv/Fm before and after the experiment. All statistics were computed in IBM © SPSS © Statistics version 22 (22.0.0.1.), USA.

## 5. Conclusions

This study shows that bryophytes may exhibit differences in site dependencies in their acclimation response in Fv/Fm to short-term temperature treatments and that using Fv/Fm as an indicator revealed those differences. The results may have implications for studies assuming similar acclimation potential between species, sites or populations of bryophytes.

## Figures and Tables

**Table 1:** One-way ANOVA of Fv/Fm and effects of altitudinal site prior to the experiment for *P. schreberi* and *R. lanuginosum*.

Variable	*Pleurozium schreberi*					*Racomitrium lanuginosum*				
	SS	*df*	MS	F	*p*	SS	*df*	MS	F	*p*
Between sites	0.43	11	0.04	1.26	n.s.	1.19	10	0.12	7.56	<0.0001
Within sites	1.11	36	0.03			0.50	32	0.02		
Total	1.54	47				1.69	42			

SS = sum of squares; *df* = degrees of freedom; MS = mean square; F = F-value; *p* = significance level; n.s. = no significant difference.

**Table 2:** Results of one-way ANOVA testing responses in Fv/Fm after four different short-term temperature treatments *ex situ* among 12 and 11 populations of *P. schreberi* and *R. lanuginosum*, respectively, originating from different altitude sites. n.s. = no significant difference.

Variable	*Pleurozium schreberi*			*Racomitrium lanuginosum*		
	*df*	F	*p*	*df*	F	*p*
Treatment	3	6.56	0.001	3	1.03	n.s.
Site	11	1.30	n.s.	10	7.50	<0.0001

**Table 3:** Results of t-test on Fv/Fm before and after short-term *ex situ* temperature treatment (day/night temperature: (1) 10/5 °C; (2) 20/10 °C; (3) 25/15 °C; (4) 30/20 °C.) in *P. schreberi* and *R. lanuginosum*. n.s. = no significant difference.

Variable	*Pleurozium schreberi*			*Racomitrium lanuginosum*		
	*df*	t	*p*	*df*	t	*p*
Fv/Fm	47	0.74	n.s.	43	−1.6	n.s.

**Table 4:** Collection sites and time of collection for *P. schreberi* and *R. lanuginosum* from Hokkaido, northern Japan. m.a.s.l. = meters above sea level.

*Pleurozium schreberi*		*Racomitrium lanuginosum*	
Site	Altitude (m.a.s.l.)	Site	Altitude (m.a.s.l.)
Mt. Oakan (43°45′N, 144°16′E)	550	Mt. Koma (42°07′N, 140°68′E)	500
29–30 June 2007	670	21–22 June 2007	550
	760		600
	865		650
	965		700
	1055		750
	1350		850
Taisetsu (43°42′N, 142°86′E)	1440		900
21 June 2007	1700		1000
	1850	Taisetsu (43°42′N, 142°86′E)	1700
Mt. Kamifurano (43°40′N, 142°67′E)	1475	21 June 2007	1910
16 June 2007	1700		

**Table 5:** Design of the temperature experiment exposing *P. schreberi* and *R. lanuginosum* to four different temperature treatments *ex situ*. Photoperiod and day/night temperature were 12 h long.

Experimental treatment	Day (12 h)	Night (12 h)
1	10 °C	5 °C
2	20 °C	10 °C
3	25 °C	15 °C
4	30 °C	20 °C

## References

[1] Wagner S., Zotz G., Salazar Allen N., Bader M.Y. (2013). Altitudinal changes in temperature responses of net photosynthesis and dark respiration in tropical bryophytes. Ann. Bot..

[2] Jägerbrand A.K., Alatalo J.M., Kudo G. (2014). Variation in responses to temperature treatments *ex situ* of the moss *Pleurozium schreberi* (Willd. Ex Brid.) Mitt. originating from eight altitude sites in Hokkaido, Japan. J. Bryol..

[3] Wagner S., Zotz G., Bader M.Y. (2014). The temperature acclimation potential of tropical bryophytes. Plant Biol..

[4] Hikosaka K., Ishikawa K., Borjigidai A., Muller O., Onoda Y. (2006). Temperature acclimation of photosynthesis: Mechanisms involved in the changes in temperature dependence of photosynthetic rate. J. Exp. Bot..

[5] Kallio P., Saarnio E. (1986). The effect on mosses of transplantation to different altitudes. J. Bryol..

[6] Furness S.B., Grime J.P. (1982). Growth rate and temperature responses in bryophytes. II. A comparative study of species of contrasted ecology. J. Ecol..

[7] Maxwell K., Johnson G.N. (2000). Chlorophyll fluorescence––A practical guide. J. Exp. Bot..

[8] Larcher W., Schulze E.D., Caldwell M.M. (1994). Photosynthesis as a tool for indicating temperature stress events. Ecophysiology of Photosynthesis. Ecological Studies 100.

[9] Csintalan Z., Proctor M.C.F., Tuba Z. (1999). Chlorophyll fluorescence during drying and rehydration in the mosses *Rhytidiadelphus loreus* (Hedw.) Warnst., *Anomodon viticulosus* (Hedw.) Hook. and Tayl. and *Grimmia pulvinata* (Hedw.) Sm. Ann. Bot..

[10] Proctor M.C.F. (2003). Experiments on the effect of different intensities of desiccation on bryophyte survival, using chlorophyll fluorescence as an index of recovery. J. Bryol..

[11] Cruz de Carvalho R.C., Branquinho C., da Silva J.M. (2011). Physiological consequences of desiccation in the aquatic bryophyte *Fontinalis antipyretica*. Planta.

[12] Kallio P., Heinonen S., Wielgolaski F.E. (1975). CO_2_ exchange and growth of *Rhacomitrium lanuginosum* and *Dicranum elongatum*. Fennoscandian Tundra Ecosystems. Part 1. Plants and Microorganisms.

[13] Glime J.M. Bryophyte Ecology. http://www.bryoecol.mtu.edu.

[14] Glime J.M. (1982). Response of *Fontinalis hypnoides* to seasonal temperature variations. J. Hattori Bot. Lab..

[15] Korpelainen H., Jägerbrand A.K., Cräutlein M.V. (2012). Genetic structure of mosses *Pleurozium schreberi* (Willd. ex Brid.) Mitt. and *Racomitrium lanuginosum* (Hedw.) Brid. along altitude gradients in Hokkaido, Japan. J. Bryol..

[16] Kallio P., Heinonen S. (1973). Ecology of *Rhacomitrium lanuginosum* (Hedw.) Brid. Rept. Kevo Subarct. Res. Stat..

[17] Gabriel R., Bates J.W. (2003). Responses of photosynthesis to irradiance in bryophytes of the Azores laurel forest. J. Bryol..

[18] Schreiber U., Schliwa U., Bilger W. (1989). Continuous recording of photochemical and non-photochemical fluorescence quenching with a new type of modulation fluorometer. Photosynth. Res..

